# The optimal therapy strategy for epidermal growth factor receptor‐mutated non‐small cell lung cancer patients with brain metastasis: A real‐world study from Taiwan

**DOI:** 10.1111/1759-7714.14423

**Published:** 2022-04-08

**Authors:** Wen‐Chien Cheng, Yi‐Cheng Shen, Chun‐Ru Chien, Wei‐Chih Liao, Chia‐Hung Chen, Te‐Chun Hsia, Chih‐Yeh Tu, Hung‐Jen Chen

**Affiliations:** ^1^ Division of Pulmonary and Critical Care, Department of Internal Medicine China Medical University Hospital Taichung Taiwan; ^2^ School of Medicine, College of Medicine, China Medical University Taichung Taiwan; ^3^ Department of Life Science National Chung Hsing University Taichung Taiwan; ^4^ Ph.D. Program in Translational Medicine, National Chung Hsing University Taichung Taiwan; ^5^ Rong Hsing Research Center for Translational Medicine National Chung Hsing University Taichung Taiwan; ^6^ Department of Radiation Oncology China Medical University Hospital Taichung Taiwan

**Keywords:** brain metastasis, epidermal growth factor receptor (*EGFR*)‐tyrosine kinase inhibitors, graded prognostic assessment for lung cancer using molecular markers (lung‐mol GPA), stereotactic radiosurgery

## Abstract

**Background:**

The treatment options for epidermal growth factor receptor (*EGFR*)‐mutated non‐small cell lung cancer (NSCLC) with brain metastases (BMs) include EGFR‐tyrosine kinase inhibitors (TKIs), stereotactic radiosurgery (SRS), whole‐brain radiotherapy, brain surgery, and antiangiogenesis therapy. As treatment options evolve, redefining optimal treatment strategies to improve survival are crucial.

**Methods:**

A total of 150 *EGFR‐*mutant NSCLC patients with BMs who received first‐ or second‐generation EGFR‐TKIs as first‐line treatment between January 2012 and October 2019 were included in this analysis.

**Results:**

After multivariate analysis, patients with the graded prognostic assessment for lung cancer using molecular markers (Lung‐mol GPA) ≥3 (hazard ratio [HR]: 0.538, 95% confidence interval [CI]: 0.35–0.83), who received afatinib or erlotinib as first‐line treatment (HR: 0.521, 95% CI: 0.33–0.82), underwent SRS therapy (HR: 0.531, 95% CI: 0.32–0.87), or were sequentially treated with osimertinib (HR: 0.400, 95% CI: 0.23–0.71) were associated with improved overall survival (OS). Furthermore, SRS plus EGFR‐TKI provided more OS benefits in patients with Lung‐mol GPA ≥3 compared with EGFR‐TKI alone in our patient cohort (44.9 vs. 26.7 months, *p* = 0.005). The OS in patients who received sequential osimertinib therapy was significantly longer than those without osimertinib treatment (43.5 vs. 24.3 months, *p* < 0.001), regardless of T790 mutation status (positive vs. negative vs. unknown: 40.4 vs. 54.6 vs.43.4 months, *p* = 0.227).

**Conclusions:**

The study demonstrated that *EGFR‐*mutant NSCLC patients with BMs could be precisely treated with SRS according to Lung‐mol GPA ≥3. Sequential osimertinib was associated with prolonged survival, regardless of T790M status.

## Graphical abstract

The results of this study provided an optimal treatment strategy for *EGFR*‐mutated non‐small cell lung cancer patients with brain metastasis. Those patients could be precisely treated with erlotinib or afatinib plus stereotactic radiosurgery according to Lung‐mol GPA ≥3. Sequential osimertinib was associated with prolonged survival in place of local brain treatment, regardless of T790M status.

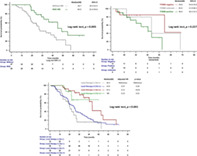

## INTRODUCTION

Non‐small cell lung cancer (NSCLC) accounts for 85% of all lung cancers, and brain metastases (BMs) are a frequent complication of NSCLC. Approximately 10%–20% of NSCLC patients had BMs at initial diagnosis, and approximately 20%–40% of NSCLC patients developed BMs during treatment.^1^ Epidermal growth factor receptor (*EGFR*) mutation is associated with approximately 40%–60% of Asian NSCLC patients and 10% of Western patients.^2^ The incidence of BMs is higher in patients with *EGFR* mutations than in those with wild‐type *EGFR*.^3^ Three generations of EGFR‐tyrosine kinase inhibitors (EGFR‐TKIs) have been approved for use in first‐line treatment for *EGFR‐*mutant NSCLC patients with BMs.^4^, ^5^, ^6^, ^7^, ^8^, ^9^ The penetration rates to cerebrospinal fluid (CSF) from plasma for gefitinib, erlotinib, afatinib, and osimertinib are 1.13, 2.77, 2.5, and 5%, respectively.^10^, ^11^, ^12^ The third‐generation EGFR‐TKI, osimertinib, had significantly better progression‐free survival (PFS), overall survival (OS), and BM response than first‐generation EGFR‐TKIs.^13^, ^14^

Despite these results, osimertinib is limited for use as a first‐line strategy in clinical practice in many countries due to its high price. Furthermore, in several studies, the OS benefit was not observed in Asian patients compared with gefitinib, erlotinib or afatinib.^7^, ^15^, ^16^, ^17^ Therefore, first‐ or second‐generation EGFR‐TKIs remain the first‐line treatment in many Asian patients with a new diagnosis of *EGFR*‐mutant NSCLC.

Because the measurable CSF concentration for first‐ or second‐generation EFGR‐TKIs in CSF is much lower than that for osimertinib, the combination of first‐ or second‐generation EGFR‐TKIs and local therapy, such as stereotactic radiosurgery (SRS), whole‐brain radiotherapy (WBRT), or brain surgery, has previously been investigated as an aggressive therapy in selective *EGFR‐*mutant NSCLC patients with BMs. A meta‐analysis of 1465 patients demonstrated that the combination of brain radiotherapy (RT) and EGFR‐TKI had better survival outcomes, especially in cases of SRS.^18^

The disease‐specific graded prognostic assessment (DS‐GPA) has previously been used for RT treatment decisions.^19^ Superduto et al. have upgraded from DS‐GPA to Lung‐mol GPA, which includes the *EGFR* and *ALK* mutation status. There were five factors in the Lung‐mol GPA, with total scores ranging from 0–4. Among patients with adenocarcinoma, the median survival varied widely from 6.9 months for those with score 0–1 to 46.8 months for those with score 3.5–4.^20^ However, few studies examining the efficacy of EGFR‐TKIs combined with local therapy have addressed the Lung‐mol GPA.^21^

Combination systemic therapy using antiangiogenesis agents and EGFR‐TKIs has also been reported to provide better intracranial control rates, longer times to intracranial progression, and fewer new BMs than EGFR‐TKIs alone.^22^, ^23^ Clinical trials (JO25567, NEJ026, and RELAY) also found that erlotinib plus vascular endothelial growth factor (VEGF) or VEGF receptor inhibitor significantly prolonged PFS among patients with *EGFR*‐mutant NSCLC.^24^, ^25^, ^26^

The majority of cancers will progress after first‐line treatment with first‐ or second‐generation EGFT‐TKIs. Sequence osimertinib in the second‐line has shown promising results for treating progressive disease, mainly due to T790M resistance mutation.^27^ However, our previous study showed that central nervous system (CNS) progression was inversely correlated with T790M mutation presence.^28^ This may not only be due to the difficulties of biopsy of CNS lesions, but recognized mechanism of pharmacokinetic resistance, a poor CSF‐to‐plasma ratio of first‐ and second‐generation EGFR‐TKIs.^29^

The identification of suitable candidates for treatment with local therapy or antiangiogenesis agents in combination with EGFR‐TKIs and the sequencing strategy with osimertinib in CNS progression remains necessary. We conducted this retrospective study with real‐world data to determine the optimal treatment strategy for *EGFR*‐mutant NSCLC patients with BMs, which may help prolong survival.

## METHODS

### Study participants

We conducted a retrospective study to analyze *EGFR*‐mutant adenocarcinoma patients with initial BMs who started EGFR*‐*TKI (gefitinib, erlotinib, or afatinib) as first‐line therapy between January 2012 and October 2019 at China Medical University Hospital. Patients who were diagnosed with BMs, confirmed by brain magnetic resonance imaging (MRI) or computed tomography, prior to initiating EGFR‐TKI therapy, were included. The exclusion criteria included patients with insufficient data for analysis, those who received treatment for less than 3 months, or those without *EGFR* mutation. The Institutional Review Board of China Medical University Hospital approved this study (CMUH 110‐REC3‐110), and informed consent was waived due to the observational and retrospective study design.

### Clinical data acquisition

The following information was extracted from electronic health records: age, sex, smoking history, Eastern Cooperative Oncology Group performance status (ECOG‐PS), the Karnofsky's index of performance status (KPS), type of sensitizing *EGFR* mutation, EGFR‐TKI treatment, PFS, the number and maximum size of brain tumors, baseline metastatic site, Lung‐mol GPA score,^20^ treatment strategies for BMs, T790M status, and the sequence osimertinib treatment. PFS was defined as the period from the initiation date of EGFR‐TKI treatment to the date of radiological or clinical evidence of progression or death. OS was defined as the time from lung cancer diagnosis to death due to any cause. The Lung‐mol GPA score included age, KPS, number of BMs, presence of extracranial metastasis, and gene mutation status. The maximum score was 4.0 (KPS 90–100: 1, age <70: 0.5, number of BM 1–4: 0.5, absence of extracranial metastasis: 1, and positive for *EGFR* mutation: 1).^20^ Additional local therapies for BMs included radiation therapy, such as WBRT or SRS, and craniotomy with brain tumor removal. Antiangiogenesis therapy, including bevacizumab or ramucirumab, was added according to the physician's assessment.

### Statistical analysis

Continuous variables are presented as the mean and standard deviation or median and interquartile range (25th and 75th percentiles). Categorical variables are expressed as percentages. Differences between continuous variables were compared using the Mann–Whitney U test or the independent *t*‐test. Differences between two independent categorical variables were compared by the chi‐square test or Fisher's exact test. A receiver operating characteristic (ROC) curve was used to determine the cutoff value of the Lung‐mol GPA. Univariate and multivariable Cox regression analyses were used to evaluate which factors are independently associated with prognosis among these patients. OS was estimated using the Kaplan–Meier method, and differences among different treatments were compared using the log‐rank test. A *p*‐value <0.05 was considered statistically significant. All statistical analyses were analyzed using MedCalc for Windows version 18.10 (MedCalc Software, Ostend, Belgium).

## RESULTS

### Patient baseline characteristics

**FIGURE 1:**
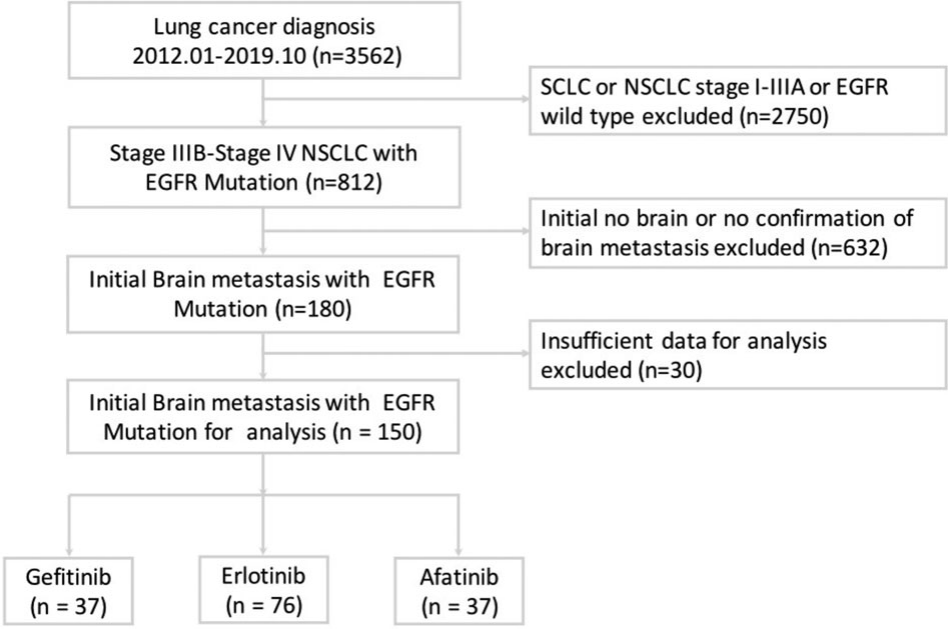
Flow diagram of patients meeting the eligibility criteria. EGFR, epidermal growth factor receptor; NSCLC, non‐small cell lung cancer; SCLC, small cell lung cancer

From January 2012 to October 2019, 3562 patients were diagnosed with lung cancer, and 812 patients with stage IIIB–IV lung adenocarcinoma received EGFR‐TKI as first‐line therapy. A total of 150 patients with initial BMs were enrolled in this study after the exclusion criteria were applied. Among these patients, 37 (37/150, 24.6%) received gefitinib, 76 (76/150, 50.6%) received erlotinib, and 37(37/150, 24.6%) received afatinib as first‐line therapy (Figure 1). The baseline characteristics of all patients are shown in Table 1. The cutoffs for Lung‐mol GPA score in our cohort were decided based on the area under the ROC curve. We supposed that scores of 3 or above indicated the prognosis was good, then the area under the ROC curve, the sensitivity, and specificity were 0.626, 0.61, and 0.58, respectively. A higher proportion of patients who received afatinib were younger than 65 years and presented with better ECOG‐PS. The proportion of patients with exon 21 L858R mutation was higher among those patients who received erlotinib. No significant differences in sex, smoking status, proportion of the neurological symptoms, maximum size of BMs, number of patients with leptomeningeal metastasis or the proportion of patients with Lung‐mol GPA ≥3 were observed among these three EGFR‐TKI treatment groups.

**TABLE 1:** Patient characteristics

	All (*n* = 150)	Gefitinib (*n* = 37)	Erlotinib (*n* = 76)	Afatinib (*n* = 37)	*p*‐value
Age ≥65 years	52 (34.7)	15 (40.5)	30 (39.5)	7 (18.9)	0.067
Male	53 (35.3)	10 (27.0)	30 (39.5)	13 (35.1)	0.430
Smoking	38 (25.3)	9 (24.3)	17 (22.4)	12 (32.4)	0.506
ECOG PS ≥2 or KPS <70	33 (22.0)	14 (37.8)	17 (22.4)	2 (5.4)	0.003
*EGFR* mutation					0.049
Del 19	76 (50.7)	21 (56.8)	34 (44.7)	21 (56.8)	–
L858R	69 (46.0)	14 (37.8)	42 (55.3)	13 (35.1)	–
Uncommon	5 (3.3)	2 (5.4)	0 (0)	3 (8.1)	–
Burden of brain metastasis					
BM symptoms	115 (77.2)	31 (83.8)	58 (76.3)	26 (72.2)	0.484
BM maximal size, cm	1.88 (1.16)	2.02 (1.18)	1.91 (1.16)	1.69 (1.15)	0.466
LM	11 (7.3)	4 (10.8)	4 (5.3)	3 (8.1)	0.557
Lung‐mol GPA ≥3	72 (48)	16 (43.2)	36 (47.4)	20 (54.1)	0.640
Treatment					
Antiangiogenesis	19 (12.7)	1 (5.3)	14 (18.4)	4 (10.8)	0.057
Local therapy modality					
WBRT^a^	80 (53.3)	23 (62.2)	33 (43.4)	24 (64.8)	0.019
SRS^b^	37 (24.6)	7 (18.9)	23 (30.2)	7 (18.9)	0.273
Brain surgery	46 (30.7)	10 (27.0)	22 (28.9)	14 (37.8)	0.643
PFS, months	10.6 (7.1–17.1)	8.4 (5.4–13.8)	10.6 (8.6–18.9)	12.1 (9.4–18.4)	0.042
Osimertinib	42 (28)	3 (8.1)	32 (42.1)	7 (18.9)	<0.001

*Note*: Continuous variables are presented as the mean (standard deviation) or median (interquartile range); categorical variables are presented as the number and percentage.

^a^ The median days between the start of EGFR‐TKI therapy and WBRT was 31 days (95% CI, 25.4–46.1 days), without statistical difference among the three groups (*p*‐value = 0.330).

^b^ The median days between the start of EGFR‐TKI therapy and SRS was 34 days (95% CI, 14.3–66.2 days), without statistical difference among the three groups (*p*‐value = 0.666).

Abbreviations: BM, brain metastasis; CI, confidence Interval; ECOG PS, Eastern Cooperative Oncology Group performance status; EGFR, epidermal growth factor receptor; KPS, the Karnofsky performance scale; LM, leptomeningeal metastases; Lung‐mol GPA, graded prognostic assessment for lung cancer with brain metastases using molecular markers; PFS, progression‐free survival; SRS, stereotactic radiosurgery; TKI, tyrosine kinase inhibitor; WBRT, whole‐brain radiotherapy.

### Treatment strategies and response assessment

After median follow‐up of 40.4 months (range 33.7–49.7 months), 97 of 150 patients had died. As shown in Table 1, the use of WBRT was lower, and the combination of antiangiogenic regimens was higher in the erlotinib group. Significantly longer PFS was noted among those patients who received afatinib as first‐line therapy (gefitinib vs. erlotinib vs. afatinib: 8.4 vs. 10.6 vs. 12.1 months, *p* = 0.042). After the failure of first‐line EGFR‐TKIs, 36 patients (36/150, 24%) patients had isolated CNS relapse, 74 patients (74/150, 49.3%) did not receive T790M mutation testing (unknown group), 46 patients (46/150, 30.6%) did not have T790M mutation (negative group), and only 30 patients (30/150, 20%) were diagnosed with T790M mutation (T790M positive group). A total of 42 patients received osimertinib as a later‐line treatment. Patients who received erlotinib as first‐line treatment had a higher rate of sequential treatment with osimertinib (gefitinib vs. erlotinib vs. afatinib: 8.1% vs. 42.1% vs. 18.9%, *p* < 0.001; Table 1).

### Clinical factors associated with survival outcomes

**FIGURE 2:**
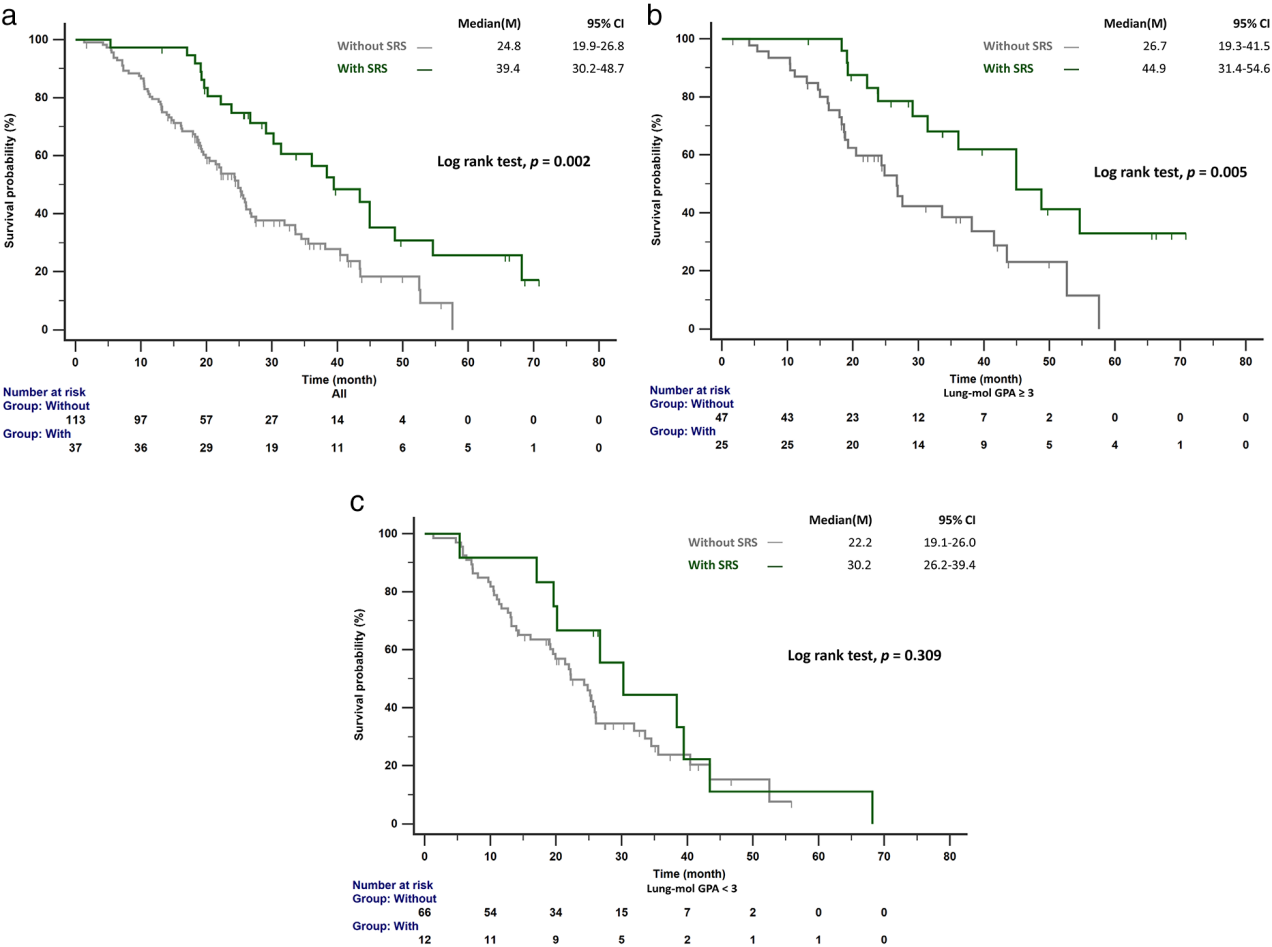
(a) Patients treated with SRS had increased median OS compared with those without SRS. (b) The median OS in patients with Lung‐mol GPA ≥3 who received EGFR‐TKI plus SRS was longer than those who received EGFR‐TKI without SRS. (c) No significant difference was observed in the median OS of patients with Lung‐mol GPA <3 who received EGFR‐TKI plus SRS and those who received EGFR‐TKI without SRS. EGFR‐TKI, epidermal growth factor receptor tyrosine kinase inhibitor; Lung‐mol GPA, graded prognostic assessment for lung cancer using molecular markers; OS, overall survival; SRS, stereotactic radiosurgery

We performed univariate and multivariate analyses of clinical factors predicting survival outcomes in *EGFR*‐mutant NSCLC patients with BMs (Table 2). Significantly longer OS was noted in patients with Lung‐mol GPA ≥3 (hazard ratio [HR]: 0.538, *p* = 0.005). Afatinib or erlotinib as first‐line treatment significantly reduced mortality compared with gefitinib (HR: 0.521, *p* = 0.004). The addition of local therapy with SRS provided patients with better outcomes (HR: 0.531, *p* = 0.014), and patients treated with EGFR‐TKI plus SRS had increased median OS than those without SRS (39.4 vs. 24.8 months; *p* = 0.002; Figure 2a). Patients were divided into two groups to identify potential differences in the benefits of additional treatment (Lung‐mol GPA ≥3 and Lung‐mol GPA <3). The median OS for patients with Lung‐mol GPA ≥3 who received EGFR‐TKI plus SRS was longer than for those treated with EGFR‐TKI without SRS (44.9 vs. 26.7 months, *p* = 0.005; Figure 2b). However, no significant difference in OS was observed between patients with Lung‐mol GPA <3 who received EGFR‐TKI plus SRS and those who received EGFR‐TKI without SRS (30.2 vs. 22.2 months, *p* = 0.309; Figure 2c). As shown in Table 2, patients who received antiangiogenesis agents appeared to have longer OS than those without antiangiogenesis treatment in the univariate analysis (HR: 0.454, *p* = 0.044). However, no significant difference in OS was observed after multivariate analysis (HR: 0.579, *p* = 0.169).

**TABLE 2:** Univariate and multivariate analysis of clinical factors associated with overall survival

	Univariate			Multivariate		
	HR	95% CI	*p*‐value	HR	95% CI	*p*‐value
Lung‐mol GPA ≥3	0.561	0.37–0.85	0.006	0.538	0.35–0.83	0.005
L858R versus Del 19	1.156	0.76–1.74	0.486	–	–	–
First‐line EGFR‐TKI						
Erlotinib versus gefitinib	0.399	0.25–0.63	<0.001	–	–	–
Afatinib versus gefitinib	0.387	0.22–0.66	<0.001	–	–	–
Afatinib versus erlotinib	0.970	0.58–1.63	0.910	–	–	–
Nongefitinib* versus gefitinib	0.395	0.26–0.60	<0.001	0.521	0.33–0.82	0.004
Local therapy						
SRS	0.454	0.28–0.75	0.001	0.531	0.32–0.87	0.014
Brain surgery	0.702	0.45–1.09	0.107	–	–	–
WBRT	1.038	0.69–1.57	0.857	–	–	–
Antiangiogenesis	0.454	0.21–0.98	0.044	0.579	0.27–1.26	0.169
Osimertinib	0.373	0.22–0.64	<0.001	0.400	0.23–0.71	0.002

Abbreviations: CI, confidence interval; EGFR, epidermal growth factor receptor; HR, hazard ratio; Lung‐mol GPA, graded prognostic assessment for lung cancer with brain metastases using molecular markers; Nongefitinib*, erlotinib or afatinib; SRS, stereotactic radiosurgery; TKI, tyrosine kinase inhibitor; WBRT, whole‐brain radiotherapy.

**FIGURE 3:**
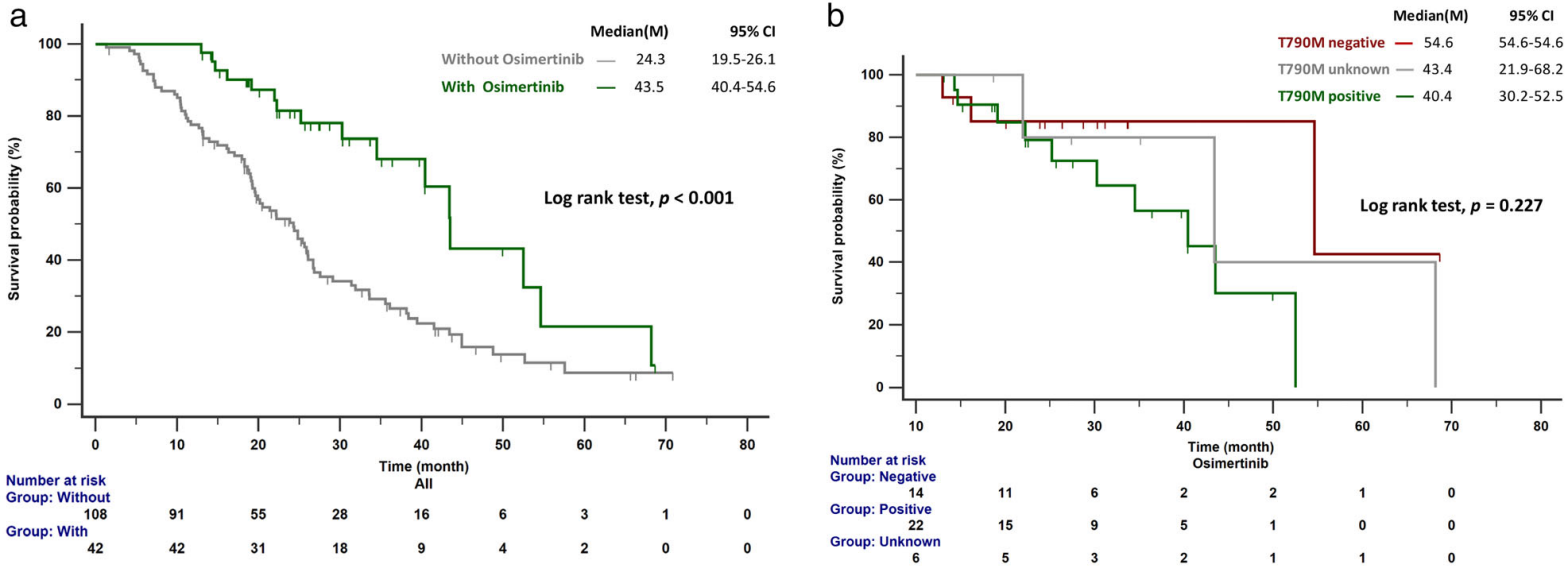
(a) The OS in patients who received sequential osimertinib was significantly longer than those without osimertinib treatment. (b) No significant difference in osimertinib treatment outcome was observed for patients with negative or unknown T790M status compared with patients with positive T790M. OS, overall survival

**FIGURE 4:**
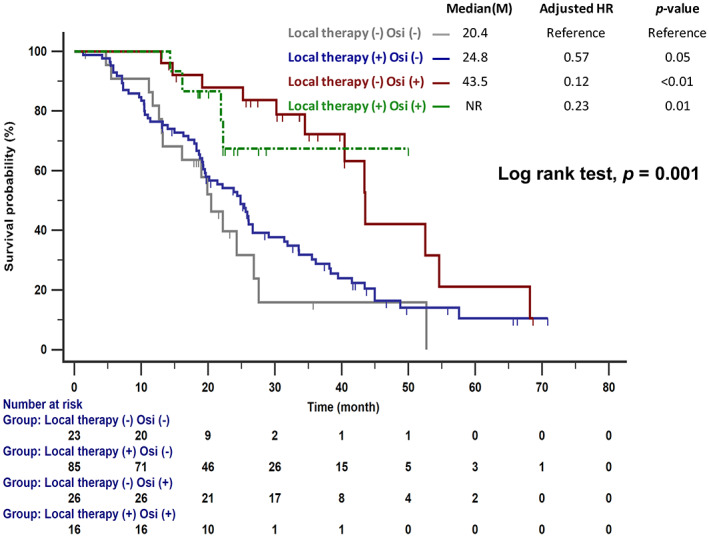
Significantly longer survival was observed in patients with sequential osimertinib therapy, regardless of additional local brain therapy, compared with those without sequential osimertinib therapy. HR, hazard ratio; Osi, osimertinib

The OS in patients who received sequential osimertinib therapy was significantly longer than in those without osimertinib treatment (43.5 vs. 24.3 months, *p* < 0.001; Figure 3a). Among those who received osimertinib, no difference in OS was observed in patients with different T790M status (positive vs. negative vs. unknown: 40.4 vs. 54.6 vs.43.4 months, *p* = 0.227; Figure 3b). Furthermore, as shown in Figure 4, significantly longer survival was observed in patients who received sequential osimertinib therapy, regardless of the use of additional local brain therapy.

## DISCUSSION

To the best of our knowledge, our study is the first to examine the effects of Lung‐mol GPA and different treatment strategies on survival in NSCLC *EGFR*‐mutant patients with BMs. We found a significantly longer OS in NSCLC *EGFR*‐mutant patients with BMs who received afatinib or erlotinib as first‐line treatment in combination with SRS. EGFR‐TKI plus SRS provided more OS benefits for patients with Lung‐mol GPA ≥3. Sequential osimertinib therapy provided OS benefits regardless of the status of T790M mutation or the addition of local brain control.

Monotherapy using first‐generation of EGFR‐TKIs can result in a 67%–88% intracranial objective response rate in EGFR‐TKI‐naïve *EGFR*‐mutant NSCLC patients.^5^, ^8^, ^9^ Several retrospective studies indicated that erlotinib is more effective than gefitinib in treating BMs due to higher levels of drug in the CSF.^10^, ^30^ Jung et al. reported that afatinib showed a superior tendency for central nervous systems (CNS)‐PFS compared with gefitinib or erlotinib.^31^ Our study reported that the initial use of afatinib or erlotinib was an independent prognostic factor for OS, which might be consistent with these studies (Table 2).

Although EGFR‐TKI monotherapy provides an acceptable intracranial response in *EGFR*‐mutant NSCLC patients, additional local treatments were investigated as aggressive treatment options to prolong intracranial control. Two meta‐analysis studies have previously reported that cranial RT (WBRT or SRS) plus TKI had higher intracranial PFS and OS than TKI therapy alone.^18^, ^32^ However, few studies have reported the benefits of cranial RT in NSCLC patients based on Lung‐mol GPA scores. Magnuson et al. indicated that patients who received upfront SRS had longer OS than those treated with WBRT or those who received EGFR‐TKI followed by RT. The survival benefit was more evident in patients with DS‐GPA 2–4 than in those with DS‐GPA 0–1.5.^21^ The current study also indicated that patients with Lung‐mol GPA ≥3 who received SRS had longer OS than those who did not receive SRS (Figure 2b). The inconsistent cutoff value of Lung‐mol GPA may be related to the different study cohort. Therefore, in NSCLC *EGFR*‐mutant patients with BMs, SRS provided better control in patients without extracranial metastases (Lung‐mol GPA ≥3). However, the addition of WBRT did not result in an OS benefit (Table 2), which was not consistent with the results reported by Wang et al.,^32^ which may be due to the influence of osimertinib on WBRT.

The dominant status of cranial RT for the treatment of *EGFR*‐mutant BMs has been challenged by the wide use of newer‐generation targeted therapies.^33^ A phase II study was conducted to evaluate the efficacy of osimertinib in patients with previously untreated BMs to avoid brain RT.^34^ Our study showed that patients treated with sequential osimertinib therapy had similar OS regardless of the use of additional local therapy (Figure 4), indicating that treatment with osimertinib could reduce the use of local therapy and avoid associated side effects. Furthermore, the current study indicated that patients with CNS‐progressed disease after first‐line EGFR‐TKI treatment who received osimertinib as sequential treatment had OS benefits regardless of T790M status. Lee et al. also reported an improvement in OS for patients who developed leptomeningeal metastases following first‐ or second‐generation EGFR‐TKI failure and were treated with subsequent osimertinib, regardless of T790M mutational status.^35^ Poor CNS penetration of first‐ and second‐generation EGFR‐TKIs has been found to be associated with pharmacokinetic resistance.^36^ The superior penetration of osimertinib through the blood–brain barrier (BBB) may explain this phenomenon.

Several limitations should be noted for this retrospective study. First, the choice of EGFR‐TKI treatment was made by the clinical physician; therefore, the number of patients in the erlotinib group was relatively larger than the numbers in the other two groups, which may be influenced by previous studies showing a higher BBB penetration rate for erlotinib. Therefore, multivariate analysis was performed to minimize potential bias. Second, the selection bias indeed existed in the NSCLC *EGFR*‐mutant patients with BMs who received SRS. The mean Lung‐mol GPA score tended to be higher without statistical significance in patients receiving SRS than those who did not receive SRS (2.91 vs 2.67; *p* = 0.06). Third, the current study did not provide intracranial PFS due to a lack of regular follow‐up brain MRI data. Fourth, 74 (49.3%) patients in our cohort did not receive T790M testing because of the difficulties of rebiopsy, especially in 36 (24%) patients with isolated CNS progression after failure of first‐line EGFR‐TKIs. Therefore, only 42 (28%) patients received sequential osimertinib therapy. Finally, financial toxicity existed among patients treated with antiangiogenesis because the medicines are not supported by health insurance in Taiwan. The number of patients receiving antiangiogenesis therapy was too small to achieve statistical significance but is worthy of further study. In spite of these limitations, our study provided the optimal treatment strategies for *EGFR*‐mutant patients with BMs in the new generation of EGFR‐TKIs era.

In conclusion, this study demonstrated that a favorable survival prognosis was identified in *EGFR‐*mutant NSCLC patients with BMs with Lung‐mol GPA ≥3 who were treated with afatinib or erlotinib in combination with SRS. Sequential osimertinib therapy may be used in place of local brain treatment, regardless of T790M status.

## CONFLICT OF INTEREST

No conflicts exist for the specified authors.
